# Development of EST Intron-Targeting SNP Markers for *Panax ginseng* and Their Application to Cultivar Authentication

**DOI:** 10.3390/ijms17060884

**Published:** 2016-06-04

**Authors:** Hongtao Wang, Guisheng Li, Woo-Saeng Kwon, Deok-Chun Yang

**Affiliations:** 1 School of Life Sciences, Yantai University, Yantai 264005, China; wht1211@gmail.com; 2 School of Pharmacy, Yantai University, Yantai 264005, China; lsyq_003@163.com; 3 Korean Ginseng Center for Most Valuable Products & Ginseng Genetic Resource Bank, Kyung Hee University, Yongin, Gyunggi-do 446-701, Korea; cosmolife@khu.ac.kr

**Keywords:** *Panax ginseng*, intron-targeting, cultivar authentication, SNP, real-time allele-specific PCR

## Abstract

*Panax ginseng* is one of the most valuable medicinal plants in the Orient. The low level of genetic variation has limited the application of molecular markers for cultivar authentication and marker-assisted selection in cultivated ginseng. To exploit DNA polymorphism within ginseng cultivars, ginseng expressed sequence tags (ESTs) were searched against the potential intron polymorphism (PIP) database to predict the positions of introns. Intron-flanking primers were then designed in conserved exon regions and used to amplify across the more variable introns. Sequencing results showed that single nucleotide polymorphisms (SNPs), as well as indels, were detected in four EST-derived introns, and SNP markers specific to “Gopoong” and “K-1” were first reported in this study. Based on cultivar-specific SNP sites, allele-specific polymerase chain reaction (PCR) was conducted and proved to be effective for the authentication of ginseng cultivars. Additionally, the combination of a simple NaOH-Tris DNA isolation method and real-time allele-specific PCR assay enabled the high throughput selection of cultivars from ginseng fields. The established real-time allele-specific PCR assay should be applied to molecular authentication and marker assisted selection of *P. ginseng* cultivars, and the EST intron-targeting strategy will provide a potential approach for marker development in species without whole genomic DNA sequence information.

## 1. Introduction

*Panax ginseng* is one of the most valuable medicinal plants in the Orient. The pharmacological effects of *P. ginseng* have been studied extensively, including improved brain function, enhanced immune system function, pain-alleviating effects, anti-tumor activity, regulated blood pressure, as well as anti-oxidative and anti-aging effects [1]. Nowadays ginseng preparations are the most popular and best-selling herbal medicines and functional foods worldwide.

As a result of increasing demand in the world market and the decrease of wild ginseng, cultivated ginseng has become the resource of ginseng preparations. Korea has been dominant in the cultivation and breeding of *P. ginseng*. A series of cultivars have been developed from three basic lines, “Jakyung”, “Chungkyung”, and “Hwangsook” by using pure line selection method, and they were registered by Korea Seed & Variety Service (www.seed.go.kr). Among these, ten cultivars including “Yunpoong”, “Gopoong”, “Sunpoong”, “Gumpoong”, “Chunpoong”, “K-1”, “Sunwon”, “Sunweon”, “Sunhyang”, and “Chungsun” are widely cultivated because they have superior agricultural traits [2]. Although these cultivars show higher yields and qualities, they are frequently mixed-cultivated with local landraces in ginseng fields, mainly due to the lack of a reliable method for authentication of ginseng cultivars. As a result, ginseng products made from different cultivars and mixed seeds are sold in the market. This not only impedes the management of ginseng cultivation, but also affects the quality control of ginseng products.

Traditional authentication methods based on phenotypic observations are error-prone because of the influence of environmental factors and similar morphological traits of ginseng cultivars. Recently, various DNA molecular marker systems—including random amplified polymorphic DNA (RAPD) [3], PCR-restriction fragment length polymorphism (RFLP) [4], inter-simple sequence repeat (ISSR) [5], amplified fragments length polymorphism (AFLP) [3], and simple sequence repeat (SSR) [6]—have been developed for Korean ginseng cultivars, but these methods are not appropriate for cultivar authentication owing to their low level of reproducibility or complicated procedures. In comparison, single nucleotide polymorphism (SNP) markers have several advantages over other marker systems, such as high abundance in genome sequences, improved results for poor quality samples, and easy automation for allele calling, and so on [7]. However, the large size and high complexity of the *P. ginseng* genome make it difficult to obtain a complete genomic sequence [8]. The objectives of this study were to (1) develop an expressed sequence tags (EST) intron-targeting strategy for SNP exploitation within *P. ginseng*; (2) construct an effective authentication and selection system for *P. ginseng* cultivars. The methodology gained in this study will provide a potential approach for marker development in species lacking whole genomic DNA sequence information.

## 2. Results

### 2.1. PCR Amplification of Expressed Sequence Tags (EST)-Derived Introns

Among 50 ESTs randomly selected from the ginseng EST database, 15 ESTs were estimated to contain introns with sizes greater than 500 bp. By prediction of intron positions from the potential intron polymorphism (PIP) database and available *P. ginseng* genomic DNA sequences, 15 pairs of EST intron-flanking primers were designed (Table 1). Polymerase chain reaction (PCR) amplifications were conducted with 15 EST intron-flanking primers and all of them amplified a single band. As expected, all of the 15 amplified amplicons showed longer sizes than the length of their corresponding EST sequences. This indicated that at least one intron was amplified for each EST sequence and the prediction of introns was indeed successful.

### 2.2. Sequence Analysis and Allele-Specific Primer Design

**Figure 1:**
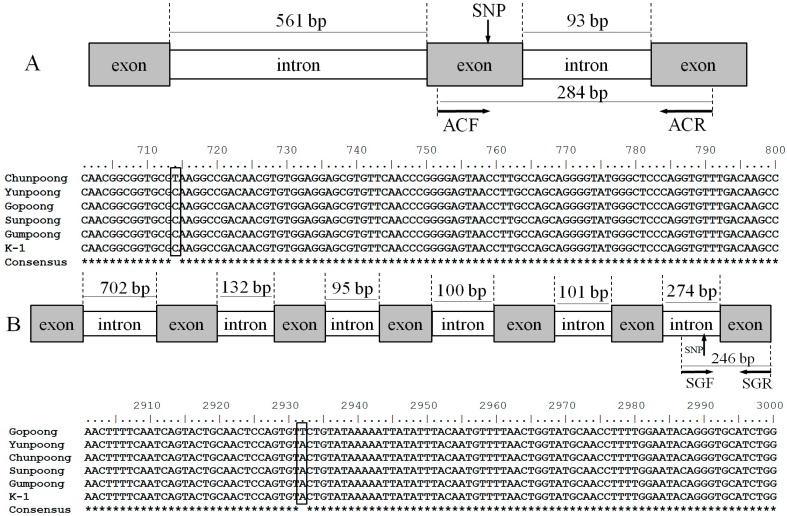

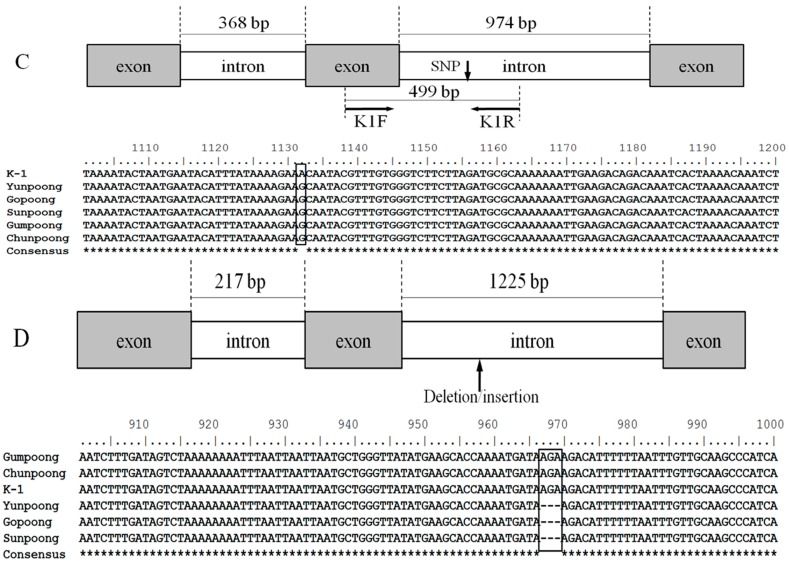
Graphic overview of the positions of exons, introns, and primer sets in target genes. (**A**) Auxin repressed protein; (**B**) Squalene epoxidase; (**C**) Cytochrome P450 (CYP71A50U); (**D**) Cytochrome P450 (CYP716A42). The framed nucleotides are DNA polymorphisms detected in this study.

Among the genomic DNA sequences of 15 targeted genes, Auxin repressed protein, Squalene epoxidase, Cytochrome P450 (CYP71A50U), and Cytochrome P450 (CYP716A42) were found polymorphic within ginseng cultivars. The polymorphic sequences of four genes were registered in GenBank with accession numbers KU342637-KU342654. The exon/intron junctions of the four genes and the polymorphic sites are shown in Figure 1. SNP sites specific to “Gopoong” and “K-1” were detected in introns of Squalene epoxidase (Figure 1B) and Cytochrome P450 (CYP71A50U) (Figure 1C), respectively. A deletion/insertion was discovered within ginseng cultivars in Cytochrome P450 (CYP716A42) (Figure 1D). Although no polymorphism was found in introns of auxin repressed protein, one SNP site specific to “Chunpoong” was exploited in its exon region (Figure 1A). Based on the SNP sites detected, primer sets ACF/ACR, SGF/SGR, and K1F/K1R were designed for specific authentication of “Chunpoong”, “Gopoong”, and “K-1”, respectively. The sequences and positions of three primer sets are shown in Figure 1 and Table 2. The substitutions of T for G, C for T, and A for T in three specific primers were introduced deliberately in order to ensure required primer specificity.

### 2.3. Allele-Specific PCR

**Figure 2:**
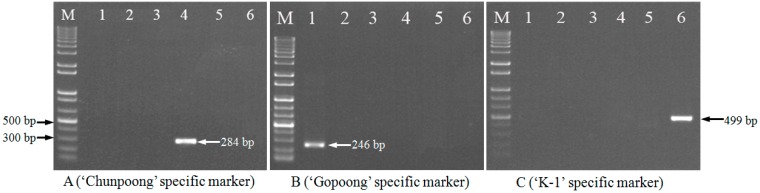
Allele-specific PCR fragment patterns using cultivar-specific primers. (**A**) ACF + ACR; (**B**) SGF + SGR; (**C**) K1F + K1R. Lane Marker (M): 1000 bp DNA ladder; lane 1: “Gopoong”; lane 2: “Yunpoong”; lane 3: “Sunpoong”; lane 4: “Chunpoong”; lane 5: “Gumpoong”; lane 6: “K-1”; lane 7: “Sunwon”; lane 8: “Sunweon”; lane 9: “Sunhyang”; lane 10: “Chungsun”.

Molecular authentication of “Chunpoong”, “Gopoong”, and “K-1” were performed by using their specific primer sets. As shown in Figure 2, “Chunpoong”, “Gopoong”, and “K-1” respectively generated their specific fragments with sizes of 284, 246 and 499 bp, but no amplicons were produced for the other cultivars. The fragment patterns of different cultivars indicated that the additional mismatches introduced in specific primers significantly reduced artificial products of the non-specific alleles. The specific 284, 246 and 499 bp PCR products were recycled and sequenced, and the sequences were confirmed to be identical to the target genomic DNA sequences. Therefore, “Chunpoong”, “Gopoong”, and “K-1” could be clearly differentiated from the other cultivars.

### 2.4. Real-Time Allele-Specific PCR Assay

**Figure 3:**
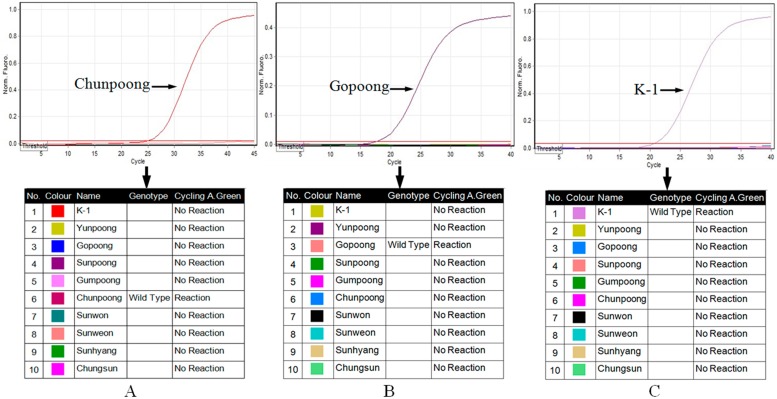
Allelic discrimination analysis of ginseng cultivars using real-time allele-specific PCR. (**A**) ACF + ACR; (**B**) SGF + SGR; (**C**) K1F + K1R.

**Figure 4:**
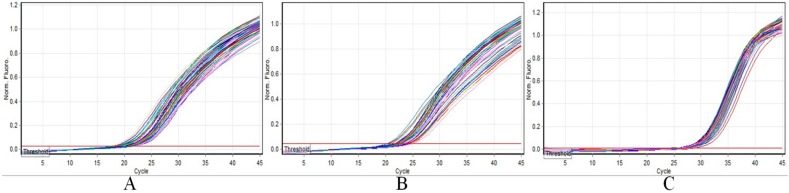
Validation of SNP markers of different ginseng cultivars using real-time allele-specific PCR. (**A**): “Chunpoong” and ACF + ACR primer pairs; (**B**) “Gopoong” and SGF + SGR pairs; (**C**) “K-1” and K1F + K1R primer pairs. Colored lines are amplification profiles of different ginseng samples.

Although SNP genotyping with allele-specific PCR is simple for the identification of ginseng cultivars, the tedious DNA isolation and agarose gel-based assay hindered its application for screening a large number of samples. To overtake this deficiency, a simple NaOH-Tris DNA isolation method and real-time allele-specific PCR with SYBR Green I fluorescent dye were combined. The unbound dye molecules fluoresce weakly at the beginning of PCR amplification, as more double-stranded PCR products are amplified, the more intensive fluorescence signal will be detected. Therefore, the presence or absence of a detected fluorescent signal indicates whether the specific allele is present in the target. As shown in Figure 3, from the amplification profiles of the ten ginseng cultivars, we can see that only “Chunpoong”, “Gopoong”, and “K-1” generated fluorescent signals during their amplification steps. After the threshold was set in the allelic discrimination analysis, “Chunpoong”, “Gopoong”, and “K-1” were regarded as the wild type while the other cultivars could not be PCR amplified. To validate the reproducibility and reliability of this authentication method, 50 samples of each cultivar were collected and analyzed, and the present method showed 100% accuracy (Figure 4). Therefore, the developed SNP markers and real-time allele-specific PCR assay is effective for field selection of ginseng cultivars.

## 3. Discussion

With the increasing demands of intra-specific markers for application in *P. ginseng* breeding and cultivar conservation, various molecular markers have been developed for ginseng cultivars, including RAPD, ISSR, PCR-RFLP, AFLP, and SSR. However, these methods are unsuitable for cultivar selection from a large number of samples. RAPD and ISSR are easily affected by a minor change of PCR conditions, and RFLP and AFLP need restriction enzyme digestion and tedious visualization protocols. Although abundant SSR polymorphisms were detected in coding and non-coding sequences of *P. ginseng*, the size differences among PCR products amplified from these cultivars are so small that silver stained polyacrylamide gel electrophoresis is usually needed.

SNP markers have proved to be simple and effective for authentication of crop cultivars, but the low levels of polymorphism within *P. ginseng* cultivars makes the development of SNP markers for cultivar identification particularly challenging. On the other hand, although a large number of sequences are obtained by genomic and transcriptomic sequencing, it is unfeasible to exploit SNPs by multiple alignments of genomic sequences or redundant EST sequences, as these sequences are usually generated from one specific cultivar. Therefore, SNP marker only could be detected by sequencing PCR products of certain loci for these ginseng cultivars, with no *a priori* expectation concerning polymorphism. Until now, SNP markers were developed only for “Chunpoong”, “Yunpoong”, and “Gumpoong” [9,10,11,12]. Previously reported SNP markers for “Chunpoong” were exploited from mitochondrial gene introns by using universal primers. The SNP marker developed in this study was located at the exon region of the auxin repressed protein gene, which may associate with the economic traits of the “Chunpoong” cultivar.

By contrast, the discovery of polymorphisms in non-coding sequences (introns) is likely to be more efficient. Introns are highly variable and evolve quickly due to experiencing much less selective pressure than exonic regions [13]. Therefore, an EST-derived intron targeting strategy is expected to yield higher polymorphism frequency than conventional PCR-based methods. Previous studies have demonstrated that intron position is highly conserved across plant species [14], thus possible introns interspersed in genes can be predicted by submitting query ginseng EST sequences to the PIP database. Intron-flanking primers then could be designed in conserved exon regions and the PCR products amplified across introns, which may exhibit length polymorphisms or SNPs. Among 15 intron-containing EST sequences, four were found to be polymorphic within ginseng cultivars, although the Chunpoong-specific SNP site was located in the exon region. The results suggest that EST intron-targeting strategy could meet the needs of marker development for ginseng cultivars.

Based on the SNP markers detected, a modified method was used to design cultivar-specific primers. The introduced artificial mismatches ensured that the SNP genotype could be easily determined by the presence or absence of a PCR product on regular agarose gels. However, high-throughput selection of ginseng cultivars from ginseng fields is difficult when using allele-specific PCR. In this study, the combination of a simple NaOH-Tris DNA isolation method and real-time allele-specific PCR with SYBR Green I fluorescent dye facilitated the fast identification and selection of ginseng cultivars. The real-time allele-specific PCR assay showed 100% accuracy and high efficiency by genotyping a large number of ginseng samples, which were collected from ginseng fields from different locations in Korea. The SNP markers of “Gopoong” and “K-1” were first reported in this study. Together with the SNP markers we developed for “Gumpoong”, “Chunpoong”, and “Yunpoong” in previous studies, our established real-time allele-specific PCR method can provide an effective authentication system, as well as a pure seed supply system, for *P. ginseng* cultivars.

Compared with previously developed molecular markers for *P. ginseng*, the SNP marker requires neither restriction enzyme digestion nor the sequence analysis of PCR products. The genotyping accuracy of the SNP marker cannot be easily achieved by a SSR marker due to its higher error rate in allele calling. Additionally, SNP genotyping can be automated in high-throughput assay formats, as it can be analyzed without requiring DNA separation by size. Therefore, the developed SNP markers, as well as the real-time allele-specific PCR assay, have clear advantages over previously used DNA markers and will be particularly useful in applications of ginseng germplasm management, including the identification of mislabeled cultivars, polygenetic analysis, and quality control in breeding and seed programs. On the other hand, although these SNP markers are efficient for cultivar discrimination, only one specific SNP per cultivar is not sufficient to be distinguished from local landrace genotypes. Multiple specific markers would be needed for the marker assisted cultivar selection among unknown genotypes. Additional efforts are underway to develop and validate SNP markers for *P. ginseng*; with more SNP markers detected in the future, they could be used not only for cultivar identification, but also for studies involving associations with a number of traits of economic value.

## 4. Materials and Methods

### 4.1. Plant Materials and DNA Isolation

Samples of ginseng plants listed in Table 3 were provided by Korean Ginseng Center for Most Valuable Products & Ginseng Genetic Resource Bank (Yongin, Korea). All voucher specimens were morphologically identified by the ginseng taxonomist, Woo-Saeng Kwon. The plant leaves were frozen in liquid nitrogen and ground into fine powders. Genomic DNA was isolated by using a plant DNA isolation kit (Exgene Plant SV mini, GeneAll, Seoul, Korea), according to the manufacturer’s instructions.

### 4.2. Prediction of Introns and Intron-Flanking Primers Design

50 EST sequences involved in three gene ontology categories (molecular function, biological process, and cellular component) were randomly selected from an EST database of *P. ginseng* [15]. Positions of introns were predicted by using the potential intron polymorphism (PIP) database [16]. EST sequences for which the corresponding genomic DNA sequences may contain introns were targeted for intron-flanking primers design. For the ESTs with available *P. ginseng* genomic DNA sequences, primers were designed according to the genomic DNA sequence. Primers flanking introns were designed by using Primer Premier 5.0 program (Premier Biosoft, Palo Alto, CA, USA).

### 4.3. PCR Amplification and Sequencing

PCR amplifications were conducted in a 20 μL volume consisting of 0.5 μM of each primer, 20 ng of template DNA, and 10 μL of 2× PreMix DNA polymerase (Genotech, Daejeon, Korea). PCR amplifications were carried out using one cycle of 4 min at 94 °C, followed by 35 cycles of 30 s at 94 °C, 30 s at a suitable annealing temperature, and 1 min extension at 72 °C with the final extension at 72 °C for 5 min. PCR products were analyzed via 1.0% agarose gel electrophoresis and visualized by ethidium bromide staining under Ultro Violet (UV). After purification through the use of a PCR Purification kit (GeneAll, Seoul, Korea), PCR products of the ten ginseng cultivars were sequenced for both forward and reverse directions on an automatic DNA sequencer (ABI PRISM 3700, Applied Biosystems, Foster City, CA, USA) by using a BigDye Terminator Cycle Sequencing Kit (Applied Biosystems, Foster City, CA, USA). DNA sequences were assembled and analyzed by using SeqMan software (DNASTAR, Madison, WI, USA).

### 4.4. Sequence Analysis and Allele-Specific Primer Design

The junctions of exons and introns were determined by comparison of ESTs and corresponding genomic DNA sequences. Multiple sequence alignments of sequences amplified from the ten ginseng cultivars were conducted using the Clustal Omega program [17]. Based on the SNP sites detected, allele-specific primers were designed by introducing additional mismatches in order to achieve the required allele specificity. Other factors (such as dimers, hairpins, or false primings) were also avoided as far as possible to minimize false-positive results.

### 4.5. Allele-Specific PCR for Authentication of P. ginseng Cultivars

Molecular authentication of three ginseng cultivars was conducted with their own specific primers and corresponding forward/reverse primers. PCR amplifications were conducted in a 20 μL volume consisting of 0.5 μM of each primer, 20 ng of template DNA, and 10 μL of 2× PreMix DNA polymerase (Genotech, Daejeon, Korea). Allele-specific PCR cycling parameters were carried out using one cycle of 4 min at 94 °C, followed by 35 cycles of 30 s at 94 °C, 30 s at a suitable annealing temperature (“Chunpoong” at 65 °C, “Gopoong” at 60 °C, and “K-1” at 60 °C), and 1 min extension at 72 °C with the final extension at 72 °C for 5 min. PCR products were analyzed via 1.0% agarose gel electrophoresis and visualized by ethidium bromide staining under UV.

### 4.6. Real-Time Allele-Specific PCR Assay for Field Selection

To facilitate the high throughput selection of cultivars from ginseng fields, a simple NaOH-Tris DNA isolation method [9] and real-time allele-specific PCR assay were combined. Real-time allele-specific PCR assays were performed on a Rotor-Gene™ 6000 machine (Corbett Life Science, Sydney, Australia). The 10 μL reaction mixture consisted of 5–10 ng DNA, 5 μM of each primer, and 5 μL 2× SYBR Green I Mastermix (SensiMixPlus SYBR, Sydney, Australia). Real-time allele-specific PCR cycling profile was carried out with 10 min of activation at 95 °C, followed by 45 cycles of a three-step thermal profile involving 10 s at 95 °C for denaturation, 15 s at the same annealing temperatures mentioned above, and 20 s at 72 °C for extension. The melting analysis condition was performed with an increase from 85 to 98 °C, rising by 1 °C at each step. Allelic discrimination analysis was used for the molecular authentication of ginseng cultivars.

## 5. Conclusions

Previous studies indicated that genetic variation was limited within these closely related ginseng cultivars, and that the lack of polymorphism prevented the molecular study of traits of agricultural importance in cultivated ginseng. This limitation can be readily overcome by exploiting more markers by the EST intron-targeting strategy. Based on the developed cultivar-specific SNP markers, the established real-time allele-specific PCR system should be applied to molecular authentication, seed purity testing, and the marker assisted selection of *P. ginseng* cultivars. Moreover, the methodology presented in this study will provide a potential approach for marker development in species lacking whole genomic DNA sequence information.

## Figures and Tables

**Table 1:** Expressed sequence tags (ESTs) for intron-flanking primers design.

EST No.	Intron-Flanking Primers (Forward (F) and Reverse (R), 5′→3′)	Annotation	Size of Target EST (bp)	Size of Amplicon (bp)
DC0_Contig 27	F–CAATCAATCACCCACCTTTG	Metallothionein-like protein	407	~1000
	R–TGACACAACAGGAAAGTCAAGG			
DC02017A01	F–TATCTCGGCTTGAAGCGTCT	Cysteine proteinase	574	~1950
	R–GTACCCCATGATCCAAATGC			
PG07020B06	F–TACCCAGTTGTGAGCGAGGAGT	Ascorbate peroxidase	406	~1300
	R–GGTCATTTCCCAGAGTAGCATTAG			
DC05020G06	F–CTTGGCAAGTTCAGGAAGATG	Auxin repressed protein	560	1215
	R–CAAACAGCAACGCTACTCGCA			
DC05003B01	F–AGAACAAACGTGAAAGGCAT	Squalene epoxidase	1751	3158
	R–GCCCTCGACTTATAGTCATACA			
DC0_contig 107	F–GATAAAGGAGTTGATGTGTGAGC	Cytochrome P450 (CYP71A50U)	1001	2353
	R–CAGGAAGGACAACACCCGAC			
DC0_contig 37	F–GCGGGATTATTGTCTTTACCT	Cytochrome P450 (CYP716A42)	987	2431
	R–TTGGGATAAATCACACCGTT			
DC05033A07	F–CGATGGAACCCTCGCTTTCTTC	Peroxiredoxin	436	~880
	R–CTCCGAGCCTGAGACAGTGAAT			
DC02026E01	F–CGAGTGGGAATTCAGAAGGAGAT	Leucine-rich repeat protein	465	~1800
	R–CACACAAGTTGTTGCTTGAGACA			
PG07002C02	F–GGCTTTGACATCGTTCGTTT	Nicotinamide adenine dinucleotide phosphate (NADP)-isocitrate dehydrogenase	450	~3000
	R–CCAAAAGCATGTCTCCCAAT			
DC04035D04	F–GCAAAGGAGCTGGTTTCATC	Glutaredoxin	343	~1700
	R–CATGGTGCAATTAACCCACA			
Contig03320	F–TTATCAGCTGCAATTCAAGC	Cycloartenol synthase	404	~2200
	R–AATCTCCATTTTCCATTTGTG			
DC02009A02	F–GGGTGTGTTCCATGTTGATAT	pathogenesis-related protein	570	~1260
	R–ATATTACAATACTGGATTTATTATC			
DC05025C05	F–GTGGGAATAAAGCACAGGAT	Epoxide hydrolase	929	~1790
	R–TTGTTGATCTCATGGGGTCT			
DC04020A06	F–GACTGATTCCGAAGAACATT	chalcone synthase	530	~1900
	R–GAAAATAAAAAAGGCACGGAA			

**Table 2:** Primer sets used for allele-specific polymerase chain reaction (PCR).

Primer Name	Nucleotide Sequence (5′→3′)	Artificial Mismatch	Amplicon Size (bp)
ACF	ATCTCCAACGGCGGT**T**CGT	G→T	284
ACR	GCTATCCTCTCCTCCTCAATG		
SGF	AGTACTGCAACTCCAG**C**GTT	T→C	246
SGR	GCCCTCGACTTATAGTCATACA		
K1F	GATAGAGAAACCATCAAAGCTG	T→A	499
K1R	AGAAGACCCACAAACGTA**A**TGT		

Underlined nucleotides are artificial mismatches introduced intentionally.

**Table 3:** Ginseng samples used in this study.

Ginseng Samples	Voucher	Location	Main Characteristics
Chunpoong	GB001	Kochang, Korea	Good root shape
Yunpoong	GB002	Kochang, Korea	High yield
Gopoong	GB003	Chuncheon, Korea	High saponin content
Sunpoong	GB004	Kochang, Korea	Early germination
Gumpoong	GB005	Kochang, Korea	High seed yield and yellow berry
K-1	GB010	Buyeo, Korea	Good root shape and well developed root hairs
Sunwon	GBD048	Daejeon, Korea	High yield
Sunweon	GBD043	Daejeon, Korea	Wrinkled leaf edges
Sunhyang	GBD058	Daejeon, Korea	High aroma constituents
Chungsun	GBD073	Daejeon, Korea	Long stem and early germination
